# Supplementary material for: HIVprotI: an integrated web based platform for prediction and design of HIV proteins inhibitors
Source: J Cheminform. 2018 Mar 9;10:12. doi: 10.1186/s13321-018-0266-y (PMC5845081; doi:10.1186/s13321-018-0266-y)
Supplement: Supplementary file 2 — Additional file 2. Source code of HIVProtI web server. [file 13321_2018_266_MOESM2_ESM.zip › HIVprotI_Source-code/hivprotI/weka-3-6-6/doc/weka/clusterers/forOPTICSAndDBScan/DataObjects/DataObject.html]

DataObject


---


|  |  |  |
| --- | --- | --- |
| **Overview** \| **Package** \| **Class** \| **Tree** \| **Deprecated** \| **Index** \| **Help** \| **Weka's home** | |  |
| PREV CLASS   **NEXT CLASS** | **FRAMES**    **NO FRAMES**     **All Classes** |  |
| SUMMARY: NESTED \| FIELD \| CONSTR \| METHOD | DETAIL: FIELD \| CONSTR \| METHOD |  |


---


## weka.clusterers.forOPTICSAndDBScan.DataObjects Interface DataObject

**All Known Implementing Classes:**: EuclidianDataObject, ManhattanDataObject

---

``` public interface DataObject ```

DataObject.java

**Version:**
:   $Revision: 1.2 $

**Author:**
:   Matthias Schubert (schubert@dbs.ifi.lmu.de), Zhanna Melnikova-Albrecht (melnikov@cip.ifi.lmu.de), Rainer Holzmann (holzmann@cip.ifi.lmu.de)

---

| **Field Summary** | |
| --- | --- |
| `static int` | `NOISE` |
| `static int` | `UNCLASSIFIED` |
| `static double` | `UNDEFINED` |


| **Method Summary** | |
| --- | --- |
| `double` | `distance(DataObject dataObject)`             Calculates the distance between dataObject and this.dataObject |
| `boolean` | `equals(DataObject dataObject)`             Compares two DataObjects in respect to their attribute-values |
| `int` | `getClusterLabel()`             Returns the clusterID, to which this DataObject belongs to |
| `double` | `getCoreDistance()`             Returns the coreDistance for this dataObject |
| `Instance` | `getInstance()`             Returns the original instance |
| `java.lang.String` | `getKey()`             Returns the key for this DataObject |
| `double` | `getReachabilityDistance()`             Returns the reachabilityDistance for this dataObject |
| `boolean` | `isProcessed()`             Gives information about the status of a dataObject |
| `void` | `setClusterLabel(int clusterID)`             Sets the clusterID (cluster), to which this DataObject belongs to |
| `void` | `setCoreDistance(double c_dist)`             Sets a new coreDistance for this dataObject |
| `void` | `setKey(java.lang.String key)`             Sets the key for this DataObject |
| `void` | `setProcessed(boolean processed)`             Marks this dataObject as processed |
| `void` | `setReachabilityDistance(double r_dist)`             Sets a new reachability-distance for this dataObject |

**Field Detail**

### UNCLASSIFIED

```
static final int UNCLASSIFIED
```

**See Also:**: Constant Field Values

---


### NOISE

```
static final int NOISE
```

**See Also:**: Constant Field Values

---


### UNDEFINED

```
static final double UNDEFINED
```

**See Also:**: Constant Field Values


**Method Detail**

### equals

```
boolean equals(DataObject dataObject)
```

:   Compares two DataObjects in respect to their attribute-values

    :   **Parameters:**: `dataObject` - The DataObject, that is compared with this.dataObject **Returns:**: Returns true, if the DataObjects correspond in each value, else returns false

---


### distance

```
double distance(DataObject dataObject)
```

:   Calculates the distance between dataObject and this.dataObject

    :   **Parameters:**: `dataObject` - The DataObject, that is used for distance-calculation with this.dataObject **Returns:**: double-value The distance between dataObject and this.dataObject

---


### getInstance

```
Instance getInstance()
```

:   Returns the original instance

    :   **Returns:**: originalInstance

---


### getKey

```
java.lang.String getKey()
```

:   Returns the key for this DataObject

    :   **Returns:**: key

---


### setKey

```
void setKey(java.lang.String key)
```

:   Sets the key for this DataObject

    :   **Parameters:**: `key` - The key is represented as string

---


### setClusterLabel

```
void setClusterLabel(int clusterID)
```

:   Sets the clusterID (cluster), to which this DataObject belongs to

    :   **Parameters:**: `clusterID` - Number of the Cluster

---


### getClusterLabel

```
int getClusterLabel()
```

:   Returns the clusterID, to which this DataObject belongs to

    :   **Returns:**: clusterID

---


### setProcessed

```
void setProcessed(boolean processed)
```

:   Marks this dataObject as processed

    :   **Parameters:**: `processed` - True, if the DataObject has been already processed, false else

---


### isProcessed

```
boolean isProcessed()
```

:   Gives information about the status of a dataObject

    :   **Returns:**: True, if this dataObject has been processed, else false

---


### setCoreDistance

```
void setCoreDistance(double c_dist)
```

:   Sets a new coreDistance for this dataObject

    :   **Parameters:**: `c_dist` - coreDistance

---


### getCoreDistance

```
double getCoreDistance()
```

:   Returns the coreDistance for this dataObject

    :   **Returns:**: coreDistance

---


### setReachabilityDistance

```
void setReachabilityDistance(double r_dist)
```

:   Sets a new reachability-distance for this dataObject

---


### getReachabilityDistance

```
double getReachabilityDistance()
```

:   Returns the reachabilityDistance for this dataObject


---


|  |  |  |
| --- | --- | --- |
| **Overview** \| **Package** \| **Class** \| **Tree** \| **Deprecated** \| **Index** \| **Help** \| **Weka's home** | |  |
| PREV CLASS   **NEXT CLASS** | **FRAMES**    **NO FRAMES**     **All Classes** |  |
| SUMMARY: NESTED \| FIELD \| CONSTR \| METHOD | DETAIL: FIELD \| CONSTR \| METHOD |  |


---
